# Development and Validation of a Self-Determination Theory-Based Measure of Motivation to Exercise and Diet in Children

**DOI:** 10.3389/fpsyg.2020.01299

**Published:** 2020-06-30

**Authors:** Giada Pietrabissa, Alessandro Rossi, Maria Borrello, Gian Mauro Manzoni, Stefania Mannarini, Gianluca Castelnuovo, Enrico Molinari

**Affiliations:** ^1^Clinical Psychology Lab, Istituto Auxologico Italiano IRCCS, Milan, Italy; ^2^Department of Psychology, Catholic University of Milan, Milan, Italy; ^3^Department of Philosophy, Sociology, Education, and Applied Psychology, Section of Applied Psychology, University of Padova, Padua, Italy; ^4^Interdepartmental Center for Family Research, University of Padova, Padua, Italy; ^5^Department of Psychology, University of Bergamo, Bergamo, Italy; ^6^Department of Psychology, eCampus University, Novedrate, Italy

**Keywords:** self-determination theory, motivation, questionnaire validation, exercise, diet, clinical psychology

## Abstract

**Objective:** To develop and test the factorial structure of a new self-determination theory–based measure of behavioral regulation in children.

**Methods:** Five hundred ninety 590 (*F* = 51.7%) children aged 7 to 11 years completed the Motivation to Exercise and Diet (MED-C) questionnaire, which comprises 16 items (eight for exercise and eight for diet) grouped into eight factors (five motivations and three needs). Psychometric testing included confirmatory factor analysis and internal consistency. Measurement invariance analyses were also performed to evaluate whether the factorial structure of the MED-C was equivalent for gender (male vs. female), age (≤9 vs. ≥10 years), and the perception of having at least one parent with overweight or obesity (yes vs. no).

**Results:** Factorial analysis confirmed an acceptable factors solution for the MED-C and a good fit to the data for both the exercise and the diet subscales assessed independently. The maximal reliability coefficient revealed good reliability for the exercise and the diet subscales. Moreover, the MED-C factor structure was invariant across group comparisons.

**Discussion:** Findings support the construct validity and reliability of the MED-C. Therefore, it represents the first validated instrument simultaneously measuring motivational regulation and psychological need satisfaction in the context of children’s exercise and diet. Considering the goodness of these results, scale percentile ranks of the total score distribution as well as the *z* score and the *T* score were provided for clinical and research purposes.

**Conclusion:** The MED-C might support the understanding of motivations and needs of children with weight problems and assist their process of behavioral change in primary and secondary prevention programs. Psychological factors represent, in fact, potential targets for interventions to increase children’s motivation to exercise and diet.

## Introduction

The prevalence of obesity in children is rising worldwide, with substantial disparities by race and ethnicity, income, education, and geographic location (World Health Organization, 2017). Among all countries in the European Union, Italy is at the top level in terms of pediatric obesity incidence and frequency. The Italian Statistic Data Center (ISTAT) reports that one million of subjects between 6 and 11 years of age are overweight or obese, with higher percentage in Southern Italy (Pecoraro et al., 2018). Children with obesity are very likely to remain obese as adults and are at risk of developing various obesity-related comorbidities, including asthma, diabetes, hypertension, and coronary heart disease, at a younger age (Llewellyn et al., 2016). Childhood obesity is also linked to lower health-related quality of life (Pulgaron, 2013) and the rising of behavioral and psychosocial problems (Hill and Silver, 1995; Borrello et al., 2015; Rankin et al., 2016; Manna and Boursier, 2018; Faccio et al., 2019; Boursier et al., 2020).

Childhood overweight and obesity are caused by the action of multiple risk factors—but it is mainly associated with various unhealthy behaviors, such as decreased physical activity and unhealthy eating habits (Toselli et al., 2014, 2015; Manzoni et al., 2018), which are significantly influenced by the family environment (Campbell and Crawford, 2001; Howe et al., 2017).

Parents are, in fact, the primary role models for children, and their behavior can positively—or negatively—influence children’s health outcomes. Children are likely to adopt the same eating habits as their parents, in particular during the first years of life (Gibson et al., 2012; Nemecek et al., 2017). Parents might also restrict highly palatable foods (e.g., sweets and fatty snacks) from their children by promoting healthy food, usually fruit and vegetables, or making use of food as a reward.

Despite the good intentions, restricting access to tasty foods focuses children’s attention on those foods and increases their desire for them (Gibson et al., 2012). In addition, some studies have found that children with restrictive parents were more likely to develop overweight later in life, especially girls (Joyce and Zimmer-Gembeck, 2009). Restriction can also lead children to eat when they are not hungry. This, in turn, could inhibit their ability to self-regulate. In addition, rewarding does not allow children to develop intrinsic motivation for healthy eating (Scaglioni et al., 2011).

Similarly, research suggests a link between parental level of physical activity encouragement, involvement/interaction, facilitation and support (Hinkley et al., 2008; Zecevic et al., 2010), and children’s exercise habits (Christofaro et al., 2019).

Lifestyle modification remains the cornerstone for sustained weight management, and motivation-based interventions have proven efficacy in promoting behavioral change (Pietrabissa et al., 2017a, b; Sorgente et al., 2017; Jackson et al., 2018; Pietrabissa, 2018).

Motivation is a determinant based on the self-determination theory (SDT) (Ryan and Deci, 2000). SDT thinks of motivation on a continuum ranging from intrinsic motivation to amotivation and proposes three universal, innate psychological needs that motivate the person to initiate a certain behavior (Ryan and Deci, 2017). These needs include *autonomy* (the need to perceive one’s self as the originator of one’s actions), *competence* (the need to feel capable to perform well in an activity), and *relatedness* (the need to feel close to and understood by important others).

In line with the SDT, the satisfaction of these needs would result in higher levels of behavioral self-determination. Further, psychological needs operate as mediators of the effects of the social context on levels of autonomous regulation (Vallerand, 1997).

Greater self-determination is reflected by high levels of *intrinsic motivation*, which is the most autonomous form of behavioral regulation, driven by inherent interest, enjoyment, and satisfaction; and lower levels of *amotivation*, defined as an absence of motivation or intention to act (Deci and Ryan, 2000). Extrinsic motivation characterizes those activities that yield specific outcomes in terms of rewards or avoided punishments. SDT conceptualizes different types of extrinsic motivation: e*xternal regulation*, when the behavior is regulated by an external incentives; *introjected regulation*, when the behavior is regulated by internalized self-judgment; *identified regulation*, the first type of the more self-determined (or autonomous) form of regulation, which occur when the behavior is explicitly recognized and valued by the individual, and *integrated regulation*, which is the most autonomous form of extrinsic motivation and arises when the behavior is fully integrated into personal values and beliefs.

Whereas external and introjected regulations may temporarily motivate change, such change is seen to produce less enduring cognitive, affective, and behavioral motivational outcomes, particularly if more autonomous forms of self-regulation are low (Deci and Ryan, 1987; Patrick and Williams, 2012; Hagger et al., 2014; Miežienë et al., 2015; Mokhtari et al., 2017).

Still, despite the importance of establishing a balanced diet accompanied by regular exercise from an early age to prevent the onset of weight problems, there is little research investigating the associations between children’s psychological needs satisfaction, their motivational to chance as defined in SDT, and their health outcomes (Borrello et al., 2015; Girelli et al., 2016; Buttitta et al., 2017).

A possible reason for this paucity of research is certainly that no comprehensive SDT-based measure of motivational regulations in children has yet been developed and tested.

In fact, researchers interested in understanding processes or mechanisms that influence change in eating behavior and exercise among children have often measured motivation by adapting a variety of scales built on other populations or domains and/or by using objective measures to address the limitations of self-report techniques (Sebire et al., 2013). Moreover, these tools mainly explore the value of SDT in understanding exercise behavior (Teixeira et al., 2012), not simultaneously accounting for the quantity and quality of motivation fostering nutritional changes. Furthermore, no validated instrument assessing psychological need satisfaction for use with children exists (Sebire et al., 2013).

To address these limitations, the present study sought to develop a new psychometrically sound instrument for a comprehensive evaluation of motivational regulations for both exercise and diet in children, also considering the role of psychological needs satisfaction in the motivational sequence.

More specifically, this study aimed to conduct a confirmatory factorial analysis (CFA) in order to examine the construct validity of the new Motivation to Exercise and Diet questionnaire in children (MED-C). The internal reliability of its subscales (exercise and diet) was also calculated, and the measurement invariance (MI) of the MED-C across several conditions was verified. The relationship between motivations (intrinsic, identified, external, introjected, amotivation) and needs (autonomy, competence, relatedness), with specific personality traits, and the presence of eating disorder symptoms was also explored.

## Materials and Methods

### Participants

Participants included 590 students from seven publicly administered institutions of higher education located in the county of Milan, Italy. Inclusion criteria were (1) being aged 7 to 11 years, (2) being native Italian speakers, (3) provision of written consent from the parents of each child involved in the research. Characteristic features of children excluded from the studies included were (1) developmental delay, (2) intellectual disability, (3) visual impairments, and (3) problems with movement and balance.

### Sample Size Calculation

Scientific literature guidelines suggest a minimum sample size of 200 observations for models of moderate complexity (Boomsma, 1983; Marsh et al., 1988; Hu and Bentler, 1999; Muthén and Asparouhov, 2002; Muthén and Muthén, 2002; Flora and Curran, 2004; Tomarken and Waller, 2005; Hoyle, 2012; Wolf et al., 2013; Brown, 2015; Kline, 2016).

Thus, a sample of 200 children was considered adequate to correctly estimate parameters of a CFA (Boomsma, 1983; Bentler and Chou, 1987; Hu and Bentler, 1999; Boomsma and Hoogland, 2001; Yu, 2002). Moreover, because the aim of the present study was to develop a new measurement tool, the “*n:q* criterion” (*n* is the number of subjects and *q* is the number of (free) model parameters to be estimated) (Hu and Bentler, 1999; Muthén and Asparouhov, 2002; Yu, 2002) was further considered, and a ratio of five subjects per parameter (5:1; *n*_minimum_ = 405) was guaranteed (Bentler and Chou, 1987; Marsh et al., 1988; Hu and Bentler, 1999; Boomsma and Hoogland, 2001; Muthén and Asparouhov, 2002; Yu, 2002; Flora and Curran, 2004; Tomarken and Waller, 2005).

### Measures

#### Demographic Survey

Prior to commencing the questionnaire, general demographic information including age, gender, body mass index–for–age percentile (BMI%), and perception to have at least one parent with overweight or obesity was collected.

Additionally, self-reported weight (in kilograms) and height (in centimeters) of the participant were used to calculate the participants’ BMI%.

#### Psychological Data

##### The Self-Administrated Psychiatric Scales for Children and Adolescents (SAFA)—P/e

The Self-Administrated Psychiatric Scales for Children and Adolescents (SAFA) is an Italian psychometric test that allows the assessment of a series of symptoms and psychiatric conditions through a total of six scales (anxiety—A, depression—D, obsessive–compulsive symptoms—O, psychogenic eating disorders—P, somatic symptoms and hypochondria—S, phobias—F, each with subscales) that can also be used separately (Cianchetti and Sannio Fancello, 2001). Each scale consists of a version for subjects aged 8 to 10 years (identified by the letter “e”) and a version for youth aged 11 to 18 years (identified by “m/s”). For the aims of the present study, the SAFA-P/e was administered. It comprises 20 items with three possible response (“true” = 2, “partly true” = 1, or “false” = 0): 10 items are intended to measure aspects of psychogenic eating disorders (bulimic behavior—P1, anorexic behavior—P2, acceptance and evaluation of one’s own body—P3), whereas the remaining 10 items assess main psychological aspects related to psychogenic eating disorders (P4—fear of maturity, perfectionism, and inadequacy). A total score of 38 indicates the presence of psychogenic eating disorders, bulimic and anorexic behaviors, and psychological risk factors. Cronbach α coefficient for the SAFA-P was 0.78 (Pellicciari et al., 2012).

##### The Kids’ Eating Disorders Survey

In order to assess the presence/absence of symptoms of disordered eating and possible eating disorders, the Italian version (Cuzzolaro et al., 1997) of the Kids’ Eating Disorders Survey (KEDS) was used. It consists of 12 items scored 0 to 2 (“yes” = 2, “no” = 1 or “don’t know” = 0), rated using two subscales (weight dissatisfaction and purging/restriction) and a global score. Scores greater than 16 are indicative of the presence of disordered eating. The KEDS showed a good internal consistency (α = 0.73) for the entire sample (*n* = 1,883) and slightly higher reliability estimates for females (α = 0.73) than for males (α = 0.70) and for children of older age (α = 0.77) than younger kids (α = 0.68) (Childress et al., 1993).

##### The Big Five Questionnaire—Children Version

The Big Five Questionnaire—Children (BFQ-C) is a 65-item measure intended to assess each of the five personality factors of energy/extraversion, agreeableness, conscientiousness, emotional instability, and intellect/openness in childhood and early adolescence (from 7 to 14 years old). Items are scored using a 5-point Likert scale ranging from 1 (almost never) to 5 (almost always). α coefficients ranged from 0.82 to 0.95 (mean = 0.88, SD = 0.04) (Barbaranelli et al., 2003).

##### The Motivation to Exercise and Diet Questionnaire—Adapted for Children

The item pool for the MED-C was developed using a three-step double-blind study procedure—already employed in other studies (Milavic et al., 2019; Pietrabissa et al., 2019).

First, three clinicians/researchers (authors GP, AR, and MB) expert in SDT and in the treatment of obesity and eating disorders independently conceived and listed approximately 15 items referring to SDT-based motivations [intrinsic (Int), identified (Id), introjected (Intr), and external (Ext) regulation, and amotivation (Am)] and needs [autonomy (A), competence (C), relatedness (R)]—giving attention to theoretical alignment and construct coverage. To ensure maximum generalization, items were not restricted to a specific target behavior (*exercise* or *diet*). According to Deci et al. (1996) and Sebire et al. (2013), integrated regulation was not included in the MED-C, as it represents an advanced form of motivation, not usually displayed by children (Deci et al., 1996; Standage and Ryan, 2012; Sebire et al., 2013).

Second, the three lists of items were merged and screened: item wordings were adjusted the target population, and redundant items were removed (Guay et al., 2010; Sebire et al., 2013). Then, a preliminary item list (32 items) was approved by the three aforementioned authors.

Third, an external collaborator submitted the list of items to 10 external experts in the field of (childhood) obesity: five psychologists, three dietitians and two pediatricians. Both the collaborator and the external experts were blind about the aim of the process. Taking into account the target population, experts were asked to list—in order of relevance—the most representative items for each type of motivation and need. An agreement higher than 80% between experts was considered adequate to retain each of the items. If agreement was reached for more than one item per dimension, experts were asked to select the most significant one. Finally, eight items (five for motivation and three for needs) were chosen. The item pool for the new MED-C was developed by applying selected items to *exercise* (*n* = 8; e.g., item 1: *I like to do sport*) as target behavior and combining these items with a set of semantically equal items targeted on *diet* (*n* = 8; e.g., item 1: *I like to eat healthy*)—see Appendix A.

Items were scaled on five-point Likert scale ranging from 0 (never) to 4 (always). Reverse scoring applied only to item 5 of each scale (Amotivation: *I don’t care about doing sports/eating healthy*).

Once item 5 of the *exercise* scale and item 5 of the *diet* scale have been reversed, the score for each scale (*exercise* or *diet*) is computed by summing the items composing each factor. Moreover, the score of each scale could be compared to the corresponding percentile rank (Table 6): a raw score under the 5° percentile rank indicates a low motivation; *z* scores and *T* scores were also provided. No overall total score (exercise *plus* diet) should be computed.

### Procedure

Between April 2018 and June 2019, participants were recruited by first contacting the head teacher from seven institutions of higher education. Once all necessary permits were obtained, administration of measures took place in the classrooms during school hours. Teachers were asked to leave the classroom to help ensure anonymity and to decrease the risk for any potential bias. During recruitment, at least one of the coauthors attended each of the classes to describe the study aims and procedures and to respond to students’ questions. Students were advised that participation was anonymous and voluntary and that they were free to discontinue the study at any time during survey completion.

Participants did not receive remuneration or incentives, but seminars were offered to parents, teachers, and students in return for their willingness and collaboration in the study—after the data collection was conducted.

Ethical approval of the study was granted by the IRCCS Istituto Auxologico Italiano Ethics Committee (ID: 03C101_2011). All procedures performed in the study were in accordance with the ethical standards of the 1964 Helsinki Declaration and its later amendments or comparable ethical standards.

Written informed consent was obtained from all the patients’ legal caregiver(s) prior to the commencement of the study.

### Statistical Analysis

This study employed quantitative approaches to examine the research questions by means of the R statistical software (version 3.5.3) (R Core Team, 2014) with the following packages: lavaan (v. 0.5-23.1097) (lavaan, 2012), psych (v. 1.8.12) (Revelle, 2018), and semTools (v. 0.4-14) (semTools Contributors, 2016).

Preliminary analyses were performed to assess potential effects of data clustering (multilevel/hierarchical) (Hedges et al., 2012; Heck and Thomas, 2015; Hox et al., 2018). In line with existing guidelines (Marsh et al., 1988; Muthén and Muthén, 2002; Brown, 2015), given the nested nature of the data (first level: subjects, second level: class, third level: school), the intraclass/intracluster correlation coefficient (ICC) was computed for each item—using maximum likelihood estimation. Moreover, as further suggested (Lai and Kwok, 2015), the design effect (DEFF) was calculated to support the ICC results. The following cutoff criteria were assumed as evidence of clustering effect: ICC > 0.050 (Kenny, 1979; Heck, 2001; Dyer et al., 2005; Thomas et al., 2005; Hayes, 2006; Geldhof et al., 2014; Brown, 2015; Grimm et al., 2017) and DEFF > 2 (Muthén and Satorra, 1995; Maas and Hox, 2005; Peugh, 2010; Lai and Kwok, 2015).

**FIGURE 1:**
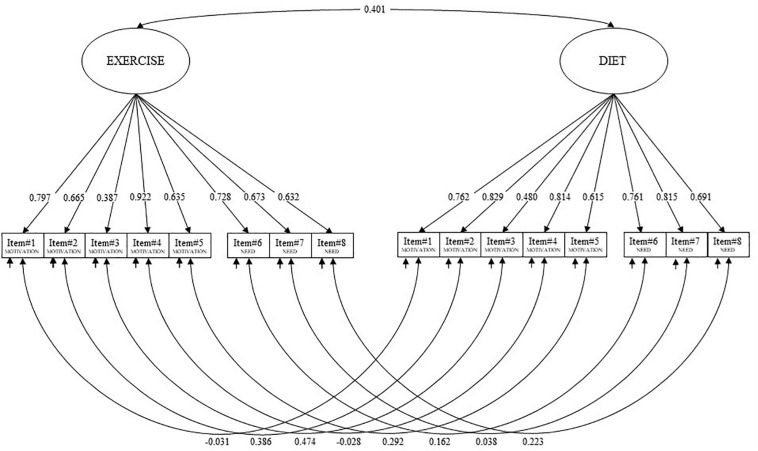
Final confirmatory factor analysis (CFA) model of the Motivation to Exercise and Diet in Children (MED-C) questionnaire.

A two-factor model was specified: eight items (five for motivation and three for needs) loaded onto the *exercise* latent factor, whereas eight semantically equal items (five for motivation and three for needs) loaded onto the *diet* latent factor (see Appendix A). The two factors were correlated. In addition, given the parallel structure of the two scales (items share similar words), correlated uniqueness (errors) was also specified between each item of a factor and its corresponding item for the other factor (Figure 1) (Kenny, 1979; Marsh, 1989; Marsh and Bailey, 1991; Kenny and Kashy, 1992; Tomás et al., 2000; Brown, 2015).

In addition, for a comprehensive evaluation of the factorial structure of the MED-C, two alternative models were further specified (see Supplementary Materials S1, S2). First, considering that correlated residuals/errors should improve model fit, main statistical analyses that could be affected by this procedure were newly performed with an equivalent model—without correlated residuals (Supplementary Material S1). Second, an alternative structure was proposed in order to address the systematic variance due to wording valence (motivation and need) while maintaining the distinction between *exercise* and *diet* (Supplementary Material S2) [e.g., (Thompson et al., 2005)].

Considering the nature of the response scale, the DWLS (diagonal weighted least square) estimator was used to assess the factorial structure of the MED-C (Muthén and Muthén, 1998–2012; Hoyle, 2012; Brown, 2015; Kline, 2016; Lionetti et al., 2016). Listwise method was use to address missing values. According to guidelines, model fit was assessed by means of the Satorra–Bentler χ^2^ statistics (S-Bχ^2^), the comparative fit index (CFI), the root mean square error of approximation (RMSEA), and the weighted root mean residual (WRMR) (Muthén and Muthén, 1998–2012; Hoyle, 2012; van de Schoot et al., 2012; Brown, 2015; Kline, 2016). Moreover, the following cutoff criteria were chosen to evaluate the goodness of fit: statistical non-significance of the χ^2^, a CFI higher than 0.95, an RMSEA lower than 0.08, and a WRMR lower than 1.00 (Muthén and Muthén, 1998–2012; Hoyle, 2012; van de Schoot et al., 2012; Brown, 2015; Kline, 2016).

Model comparisons were performed to exclude structures other than the one formulated in the *a priori* hypothesis for the MED-C (Boffo et al., 2012; Milavic et al., 2019; Rossi and Mannarini, 2019).

Specifically, (A) a two-factor model without correlated errors, (B) a two-independent-factors, model, (C) a single-factor model, (D) a second-order model (hierarchical), and (E) a bifactor model (hierarchical) were tested.

Model evaluations were performed by using the test differences in three fit indices, with the following criteria as cutoffs for model equality: DIFFTEST (equal to Δχ^2^, *p* > 0.050), ΔCFI (<0.010), and ΔRMSEA (<0.015) (Cheung and Rensvold, 2002; Millsap, 2012; van de Schoot et al., 2012; Brown, 2015; Rossi and Mannarini, 2019). The crossing of the cutoff of two of three of these indices is evidence of model inadequateness.

Moreover, considering that the MED-C is a new scale, items ability to discriminate subjects with low or high motivation for *exercise* and *diet* were tested (Ebel, 1965; Chiorri, 2011). According to existing guidelines, item–total correlation (adjusted) was computed (Howell, 2013; Pallant, 2013; Tabachnick and Fidell, 2014). Moreover, item discriminant power (IDP) for typical performance items (e.g., Likert scale) was also carried out. More in detail, the maximum total score and quartile rank for each subject were calculated. Subsequently, a series of independent-samples *t* test—and their effect size (Cohen *d*) (Cohen, 1988)—were calculated to assess item discriminating power by using the total score of the scale as dependent variable and its lowest and highest quartile as grouping variable (Ebel, 1965; Chiorri, 2011).

Because of possible differences in the magnitude of factor loadings, maximal reliability (MR) coefficient (Raykov, 2012) was chosen as measure of internal consistency of each factor—instead of Cronbach α (Raykov, 2011, 2012; Raykov and Marcoulides, 2011; Barbaranelli et al., 2014).

Measurement invariance analyses were also performed to evaluate whether the factorial structure of the MED-C was invariant between: (A) gender (male vs. female) and (B) age (≤9 vs. ≥10 years—according to the median split technique [e.g., (Ratti et al., 2017)], and (C) perception to have at least one parent with overweight or obesity (yes vs. no) (Vandenberg and Lance, 2000). According to Meredith (1993) and Millsap (2012), model structure was tested on each sample independently (Meredith, 1993). If model fit was adequate in each sample, four nested models were sequentially specified and constrained to equality: the factorial structure (Model 1: configural invariance); the factorial structure and item factor loadings (Model 2: metric invariance); the factorial structure, item factor loadings, and item thresholds (Model 3: scalar invariance); the factorial structure, item factor loadings, item thresholds, and latent means (Model 4: scalar invariance); (Meredith, 1993; Vandenberg and Lance, 2000; Millsap, 2012; van de Schoot et al., 2012). Measurement invariance was assessed by using aforementioned test differences for model comparisons (Cheung and Rensvold, 2002; Millsap, 2012; van de Schoot et al., 2012; Brown, 2015; Rossi and Mannarini, 2019).

Convergent validity was assessed with the Pearson correlation coefficient (Tabachnick and Fidell, 2014) and interpreted using the Cohen benchmarks: *r* < 0.10, trivial; *r* from 0.10 to 0.30, small; *r* from 0.30 to 0.50, moderate; *r* > 0.50, large (Cohen, 1988).

## Results

### Sample Characteristics

Of 590 participants, 305 were female (51.7%), and 285 were male (48.3%). The mean age of the sample was 9.54 (SD = 1.003; range 7–11 years). The average BMI% was 58.44 (SD = 29.66; range 1st–99th percentile); more in detail, 26 were underweight, 418 had a normal weight, 91 were overweight, and 55 had obesity.

### Preliminary Analysis—Testing for Multilevel Data Structure

As reported in Table 1, only six items (37.5%) of the 16 composing the MED-C revealed an ICC higher than the recommend threshold—showing that the majority of the items (*n* = 10; 62.5%) of the MED-C had no clustering effect of the class (second level) or of the school (third level). Moreover, DEFF showed that only a single item (item 7—*diet*) showed a clustering effect, whereas all the other item had a design effect lower than 2. Considering these results, non-multilevel statistics were further run.

**TABLE 1:** Items’ multilevel properties: within and between variance, ICC, and design effect.

	Class * school *n* clusters = 43 (2nd level); 7 (3rd level) mean_subj_ per cluster = 13.68			
	Within variance	Between variance	ICC	Design effect
**Exercise**				
Item 1	0.760	0.040	0.050	1.643
Item 2	0.909	0.075	**0.076**	1.980
Item 3	1.872	0.089	0.045	1.584
Item 4	0.938	0.061	**0.061**	1.785
Item 5	0.616	0.007	0.011	1.144
Item 6	1.266	0.104	**0.076**	1.976
Item 7	1.236	0.018	0.014	1.185
Item 8	1.599	0.055	0.033	1.428
**Diet**				
Item 1	1.289	0.068	0.050	1.640
Item 2	1.165	0.063	**0.051**	1.660
Item 3	1.755	0.113	**0.060**	1.778
Item 4	1.325	0.064	0.046	1.592
Item 5	0.956	0.007	0.007	1.093
Item 6	1.653	0.082	0.047	1.608
Item 7	1.359	0.122	**0.082**	**2.059**
Item 8	1.387	0.031	0.022	1.281

*Values higher than the recommended threshold are in bold font (ICC > 0.050; design effect > 2).*

### Structural Validity

First, to ensure the goodness of the structural validity of each scale, model fits for *exercise* and *diet* scale were assessed independently. Then, the model fit of the two join scales was assessed.

The *exercise* scale of the MED-C showed a good fit to the data. The χ^2^ statistic resulted to be non-statistically significant [S-Bχ^2^(20) = 22.750, *p* = 0.302 *ns*], and all the other fit indices revealed a good fit to the data: the CFI = 0.999, the RMSEA = 0.016; 90% confidence interval (CI) = 0.000–0.041; *p*(RMSEA < 0.05) = 0.992, the WRMR = 0.616. All the items’ loadings were statistically significant and ranged from 0.392 (item 4PA) to 0.904 (item 3PA), with a mean equal to 0.680 and an SD equal to 0.151.

The *diet* scale of the MED-C showed a good fit to the data. Despite that the χ^2^ statistic resulted to be statistically significant [S-Bχ^2^(20) = 54.500, *p* < 0.001], all the other fit indices revealed a good fit to the data: the CFI = 0.996, the RMSEA = 0.057; 90% CI = 0.039–0.075; *p*(RMSEA < 0.05) = 0.252, the WRMR = 0.953. As reported in Table 2, all the items’ loadings were statistically significant and ranged from 0.493 (item 4—*diet*) to 0.835 (item 2—*diet*), with a mean equal to 0.730 and an SD equal to 0.118.

**TABLE 2:** Item descriptive statistics, item discriminant power (IDP), item–total adjusted correlation (IT-TOT), and confirmatory factor analysis (CFA).

	Descriptive statistics					IDP		IT-TOT	CFA	
	Mean	Median	SD	SK	K	*t*	*d*	*r*_Adj_	|λ|	*R*^2^
**Exercise**										
Item 1	3.336	4	0.895	−1.440	2.067	−14.906	1.799	0.635	0.797	0.635
Item 2	3.218	4	0.992	−1.213	0.903	−15.026	1.825	0.527	0.665	0.442
Item 3	2.009	2	1.403	−0.008	−1.228	−17.050	2.040	0.313	0.378	0.143
Item 4	3.419	4	1.000	−1.917	3.166	−15.442	1.895	0.687	0.922	0.850
Item 5*	0.273	0	0.790	3.253	10.461	7.782	0.946	0.419	0.635	0.404
Item 6	3.189	4	1.171	−1.465	1.249	−18.895	2.286	0.584	0.728	0.530
Item 7	3.053	3	1.121	−1.182	0.701	−16.245	1.986	0.555	0.673	0.453
Item 8	2.771	3	1.287	−0.812	−0.422	−20.511	2.500	0.522	0.632	0.399
**Diet**										
Item 1	2.640	3	1.171	−0.520	−0.430	−22.048	2.574	0.667	0.762	0.580
Item 2	3.063	3	1.110	−1.057	0.353	−20.899	2.389	0.692	0.829	0.687
Item 3	1.532	1	1.369	0.386	−1.070	−14.806	1.751	0.405	0.480	0.230
Item 4	2.814	3	1.180	−0.765	−0.273	−20.738	2.398	0.693	0.814	0.663
Item 5*	0.532	0	0.982	1.976	3.302	11.462	1.317	0.440	0.615	0.378
Item 6	2.375	2	1.319	−0.398	−0.895	−23.322	2.758	0.678	0.761	0.579
Item 7	2.516	3	1.217	−0.513	−0.546	−21.764	2.510	0.725	0.815	0.664
Item 8	2.170	2	1.192	−0.215	−0.672	−21.088	2.467	0.619	0.691	0.477

*Reverse scoring. In the CFA columns, absolute values of standardized factor loading (|λ|) are reported.

Finally, the MED-C was tested, and it showed a good fit to the data. Despite that the χ^2^ statistic resulted to be statistically significant [S-Bχ^2^(95) = 139.769, *p* = 0.002], all the other fit indices revealed a good fit to the data: the CFI = 0.997, the RMSEA = 0.030; 90% CI = 0.019–0.040; *p*(RMSEA < 0.05) = 1, the WRMR = 0.872. As reported in Table 2, all the items’ loadings were statistically significant and ranged from 0.378 (item 4) to 0.922 (item 3), with a mean equal to 0.700 and an SD equal to 0.137.

### Psychometrics Properties

The IDP analysis showed that 16 items of the MED-C discriminated well between subjects with low and high motivation to *exercise* and *diet* (Table 2). The discrimination parameter *t*_i_ ranged from |7.782| (item 5—*exercise*) to |23.322| (item 6—*diet*), with an associated effect size (Cohen *d*) ranging from 0.946 to 2.758, respectively. Also, the item–total correlation (adjusted) revealed a strong association between each item and the MED-C total score.

Moreover, the two first-order factor solution was compared with different competing models that could also explain the MED-C factorial structure (Muthén and Muthén, 1998–2012; Brown, 2015; Rossi and Mannarini, 2019). As reported in Table 3, model comparisons revealed the superiority of the original proposed solution: two related first-order factor models with correlated uniqueness, accounting for two different dimensions. Consequently, this factorial solution was chosen to perform following analysis.

**TABLE 3:** Model comparison.

	S-Bχ^2^(*df*)	RMSEA	CFI	Comparison	DIFF-TEST	|ΔRMSEA|	|ΔCFI|
Model 1: two related factors—with correlation of residuals	139.760** (95)	0.030	0.997				
Model 2: two related factors—no correlation of residuals	288.593*** (103)	0.059	0.987	2 vs. 1	148.82*** (8)	0.029	0.010
Model 3*:* two-independent-factor model	1,534.712*** (104)	0.163	0.899	3 vs. 1	1,394.90*** (9)	0.133	0.098
Model 4: single-factor model	1,683.750*** (104)	0.171	0.889	4 vs. 1	1,544.00*** (9)	0.141	0.108
Model 5: second-order model	Not identified	—	—	5 vs. 1	—	—	—
Model 6: bifactor model	No convergence	—	—	6 vs. 1	—	—	—

***p < 0.001. S-Bχ^2^, Satorra–Bentler scaled chi-square test; *df*, degrees of freedoms; |Δ(…)|, absolute value of the differences between indices; RMSEA, root mean square error of approximation; CFI, comparative fit index.

Reliability analysis revealed satisfying results. Indeed, for the *exercise* subscale, the MR was equal to 0.876, and for the *diet* subscale, the MR was equal to 0.901.

As shown in Table 5, small to moderate correlations were found between the *exercise* subscale and the BFQ-C subscales (energy/EXTR: *r* = 0.400, *p* < 0.001; AGREE: *r* = 0.214, *p* < 0.001; COSC: *r* = 0.299, *p* < 0.001; INT/OPN *r* = 0.302, *p* < 0.001). Small correlations were found between the *exercise* subscale and both the KEDS (total score: *r* = 0.161, *p* < 0.001; W_DISS: *r* = 0.150, *p* < 0.001) and the SAFA (BULIM: *r* = −0.092, *p* = 0.026; PERFE: *r* = 0.159, *p* < 0.001; INAD: *r* = −0.095, *p* = 0.022).

In line with previous results, small to moderate correlations were found between the *diet* subscale and the BFQ-C subscales (energy/EXTR: *r* = 0.164, *p* < 0.001; AGREE: *r* = 0.243, *p* < 0.001; COSC: *r* = 0.332, *p* < 0.001; INT/OPN *r* = 0.343, *p* < 0.001). Small correlations were found between the *diet* subscale and both the KEDS (total score: *r* = 0.144, *p* < 0.001; W_DISS: *r* = 0.148, *p* < 0.001; PURG: *r* = 0.108, *p* = 0.009) and the SAFA (BULIM: *r* = −0.112, *p* = 0.007; AN: *r* = 0.136, *p* = 0.001; PERFE: *r* = 0.130, *p* = 0.002; INAD: *r* = −0.160, *p* < 0.001).

### Measurement Invariance

#### Gender (Male vs. Female)

##### Configural invariance

The configural invariance model showed good model fit indices: S-Bχ^2^(190) = 225.56, CFI = 0.998, and the RMSEA = 0.027, suggesting that the factor structure was similar between males and females.

##### Metric invariance

The metric invariance model well-fitted the data: S-Bχ^2^(204) = 232.68, CFI = 0.998, and the RMSEA = 0.023. Non-significant decreases in fit indices were found [DIFTEST (14) = 7.125, *p* = 0.930, |ΔRMSEA| = 0.004, |ΔCFI| = 0.000], indicating that items were equivalently related to the latent factor irrespectively of gender.

##### Scalar invariance

The scalar invariance model showed good model fit indices: S-Bχ^2^(250) = 277.54, CFI = 0.998, and the RMSEA = 0.021. Non-significant decreases in fit indices were found [DIFTEST (46) = 44.858, *p* = 0.520, |ΔRMSEA| = 0.002, |ΔCFI| = 0.000], suggesting that males and females had the same expected item response at the same absolute level of the trait.

##### Latent means invariance

The latent mean invariance model well-fitted the data: S-Bχ^2^(252) = 366.56, CFI = 0.992, and the RMSEA = 0.042. Significant decreases in fit indices were found [DIFTEST (2) = 89.015, *p* < 0.001, |ΔRMSEA| = 0.021, |ΔCFI| = 0.006], suggesting that males and females had not the same expected latent mean of the traits.

#### Age (Median Slit Technique: ≤9 vs. ≥10 Years)

##### Configural invariance

The configural invariance model showed good model fit indices: S-Bχ^2^(190) = 239.19, CFI = 0.987, and the RMSEA = 0.032, suggesting that the factor structure was similar between different age-related groups.

##### Metric invariance

The metric invariance model well-fitted the data: S-Bχ^2^(204) = 305.89, CFI = 0.993, and the RMSEA = 0.044. In this case, non-significant decreases in fit indices were found [DIFTEST (14) = 66.699, *p* < 0.001, |ΔRMSEA| = 0.012, |ΔCFI| = 0.003], indicating that items were equivalently related to the latent factor between groups.

##### Scalar invariance

The scalar invariance model showed good model fit indices: S-Bχ^2^(250) = 352.21, CFI = 0.993, and the RMSEA = 0.040. Non-significant decreases in fit indices were found [DIFTEST (46) = 46.330, *p* = 0.459, |ΔRMSEA| = 0.004, |ΔCFI| = 0.000], suggesting that the two groups had the same expected item response at the same absolute level of the trait.

##### Latent means invariance

The latent mean invariance model showed good model fit indices: S-Bχ^2^(252) = 366.58, CFI = 0.993, and the RMSEA = 0.042. Non-significant decreases in fit indices were found [DIFTEST (2) = 14.366, *p* < 0.001, |ΔRMSEA| = 0.002, |ΔCFI| = 0.000], suggesting that the two groups had the same expected latent mean of the traits.

#### Perception to Have at Least One Parent With Overweight or Obesity (Yes vs. No)

##### Configural invariance

The configural invariance model showed good model fit indices: S-Bχ^2^(190) = 245.75, CFI = 0.996, and the RMSEA = 0.034, suggesting that the factor structure was similar between children who perceive to have at least one parent with overweight/obesity and children who did not.

##### Metric invariance

The metric invariance model well-fitted the data: S-Bχ^2^(204) = 254.82, CFI = 0.997, and the RMSEA = 0.031. Non-significant decreases in fit indices were found [DIFTEST (14) = 9.065, *p* = 0.827, |ΔRMSEA| = 0.003, |ΔCFI| = 0.001], indicating that items were equivalently related to the latent factor between groups.

##### Scalar invariance

The scalar invariance model showed good model fit indices: S-Bχ^2^(250) = 304.28, CFI = 0.996, and the RMSEA = 0.029. Non-significant decreases in fit indices were found [DIFTEST (46) = 49.461, *p* = 0.337, |ΔRMSEA| = 0.002, |ΔCFI| = 0.001], indicating that items were equivalently related to the latent factor irrespectively of children’s perception to have at least one parent with overweight/obesity and their counterpart.

##### Latent means invariance

The latent mean invariance model still fitted data well: S-Bχ^2^(252) = 304.89, CFI = 0.996, and the RMSEA = 0.028. Non-significant decreases in fit indices were found [DIFTEST (2) = 0.611, *p* = 0.737, |ΔRMSEA| = 0.001, |ΔCFI| = 0.000], suggesting that the two groups had the same expected latent mean of the traits.

### Normative Scores of the MED-C

The fit statistics are presented in Table 4. Finally, considering the goodness of these results and the need to compare the score between the MED-C dimensions (*exercise* and *diet*), scale percentile ranks of the total score distribution as well as the *z* score and the *T* score were provided to facilitate the use of the questionnaire in clinical settings (Table 6).

**TABLE 4:** Measurement invariance.

	S-Bχ^2^(*df*)	RMSEA	CFI	DIFF-TEST	*p*(DIFFTEST)	|ΔRMSEA|	|ΔCFI|
**Gender**							
Model “male” (*n* = 285)	102.46 (95)	0.018	0.999				
Model “female” (*n* = 305)	123.10 (95)	0.033	0.995				
Configural inv.	225.56 (190)	0.027	0.998				
Metric inv.	232.68 (204)	0.023	0.998	7.125 (14)	*p* = 0.930	0.004	0.000
Strict inv.	277.54 (250)	0.021	0.998	44.858 (46)	*p* = 0.520	0.002	0.000
Mean inv.	366.56 (252)	0.042	0.992	89.015 (2)	*p* < 0.001	0.021	0.006
**Age**							
Model “≤9 y.o.” (*n* = 269)	117.26 (95)	0.032	0.996				
Model “≥10 y.o.” (*n* = 321)	121.92 (95)	0.032	0.997				
Configural inv.	239.19 (190)	0.032	0.997				
Metric inv.	305.89 (204)	0.044	0.993	66.699 (14)	*p* < 0.001	0.012	0.004
Strict inv.	352.21 (250)	0.040	0.993	46.330 (46)	*p* = 0.459	0.004	0.000
Mean inv.	366.58 (252)	0.042	0.993	14.366 (2)	*p* < 0.001	0.002	0.000
**Parent(s) with obesity**							
Model “yes” (*n* = 225)	98.88 (95)	0.014	0.999				
Model “no” (*n* = 365)	146.87 (95)	0.041	0.994				
Configural inv.	245.75 (190)	0.034	0.996				
Metric inv.	254.82 (204)	0.031	0.997	9.065 (14)	*p* = 0.827	0.003	0.001
Strict inv.	304.28 (250)	0.029	0.996	49.461 (46)	*p* = 0.337	0.002	0.001
Mean inv.	304.89 (252)	0.028	0.996	0.611 (2)	*p* = 0.737	0.001	0.000

*S-Bχ^2^, Satorra–Bentler scaled chi-square test; *df*, degrees of freedoms; |Δ(…)|, absolute value of the differences between indices; RMSEA, root mean square error of approximation; CFI, comparative fit index.*

**TABLE 5:** Convergent validity.

		Mean	SD	*r*_(__Exercise__)_	*r*_(__Diet__)_
	**MED-C**				
1	Exercise	24.56	5.683	—	
2	Diet	20.27	6.733	0.342**	—
	**BFQ-C**				
3	Energy–extraversion	30.07	3.712	0.400**	0.164**
4	Agreeableness	30.61	4.366	0.214**	0.243**
5	Conscientiousness	30.28	4.275	0.299**	0.332**
6	Emotional–instability	23.30	5.091	−0.072^§^	−0.050^§^
7	Intellect–openness	29.94	4.238	0.302**	0.343**
	**KEDS**				
8	Total	12.70	2.549	0.161**	0.144**
9	Body dissatisfaction	–0.49	1.292	0.054^§^	0.004^§^
10	Weight dissatisfaction	7.87	2.112	0.150**	0.148**
11	Purging/restricting	2.95	0.263	0.053^§^	0.108**
12	Binge eating	0.89	0.403	0.063^§^	0.003^§^
	**SAFA**				
13	Total	14.32	6.342	−0.042^§^	–0.030
14	Bulimic behavior	2.01	2.079	−0.092*	−0.112**
15	Anorexic behavior	2.32	1.681	0.051^§^	0.136**
16	Acceptance and evaluation of ones’ body	1.63	1.888	−0.049^§^	−0.014^§^
17	Fear of maturity	2.84	1.959	−0.080^§^	−0.048^§^
18	Perfectionism	3.73	1.516	0.159**	0.130**
19	Inadequacy	1.79	1.630	−0.095*	−0.160**

^§^*p* > 0.050 *ns*, *p < 0.050, **p < 0.001.

**TABLE 6:** Raw scores, frequencies of the raw score, percentiles of the distribution of the raw score, *Z* scores and *T* scores for motivation to *exercise* and *diet* scales.

Exercise					Diet				
Raw score	Freq.	Percentile rank	*Z* score	*T* score	Raw score	Freq.	Percentile rank	*Z* score	*T* score
0	2	1	−4.32	6.78	0	3	1	−3.01	19.89
1	1	1	−4.15	8.54	1	2	1	−2.86	21.38
2	1	1	−3.97	10.30	2	3	2	−2.71	22.86
3	1	1	−3.79	12.05	3	2	2	−2.57	24.35
4	1	2	−3.62	13.81	4	6	3	−2.42	25.83
5	1	2	−3.44	15.57	5	2	3	−2.27	27.32
6	2	2	−3.27	17.33	6	4	4	−2.12	28.80
7	2	2	−3.09	19.09	7	10	5	−1.97	30.29
8	1	3	−2.91	20.85	8	6	6	−1.82	31.77
9	2	3	−2.74	22.61	9	6	7	−1.67	33.26
10	4	3	−2.56	24.37	10	8	9	−1.53	34.74
11	1	4	−2.39	26.13	11	8	10	−1.38	36.23
12	2	4	−2.21	27.89	12	21	12	−1.23	37.71
13	4	4	−2.03	29.65	13	11	15	−1.08	39.20
14	3	5	−1.86	31.41	14	12	17	−0.93	40.68
15	12	6	−1.68	33.17	15	21	20	−0.78	42.17
16	9	8	−1.51	34.93	16	32	24	−0.63	43.65
17	15	10	−1.33	36.69	17	31	29	−0.49	45.14
18	12	12	−1.16	38.45	18	34	35	−0.34	46.62
19	20	15	−0.98	40.21	19	24	40	−0.19	48.11
20	20	18	−0.80	41.97	20	28	44	−0.04	49.59
21	20	22	−0.63	43.73	21	29	49	0.11	51.08
22	30	26	−0.45	45.49	22	36	54	0.26	52.56
23	51	33	−0.28	47.25	23	49	61	0.40	54.05
24	31	40	−0.10	49.01	24	33	68	0.55	55.53
25	38	45	0.08	50.77	25	30	74	0.70	57.02
26	52	53	0.25	52.53	26	30	79	0.85	58.50
27	35	60	0.43	54.29	27	27	83	1.00	59.99
28	67	69	0.60	56.05	28	27	88	1.15	61.47
29	44	78	0.78	57.80	29	20	92	1.30	62.96
30	37	85	0.96	59.56	30	11	94	1.44	64.44
31	32	91	1.13	61.32	31	9	96	1.59	65.93
32	37	96	1.31	63.08	32	15	98	1.74	67.41

## Discussion

This study provided evidence for the performance and theoretical alignment of the novel MED-C questionnaire for assessing physical activity and healthy-eating motivation in a general population of children aged 7 to 11 years.

Preliminary analyses demonstrated that the hierarchical/multilevel data structure of the study sample did not influence the majority of the items of the MED-C, thus providing support for the non-dependence of the items from the children’s social context (Hedges et al., 2012; Heck and Thomas, 2015; Hox et al., 2018). The motivation to exercise and motivation to diet dimensions of the MED-C questionnaire can therefore be considered free from data clustering.

Confirmatory factorial analysis confirmed that all the 16 items of the MED-C loaded onto the designated latent factor (Figure 1) and that the MED-C—as well as its two subscales assessed independently—possesses good structural validity with excellent fit indices. Factor loading showed a strong relationship between the items and the corresponding latent factor, suggesting each item to be a valid representative of the construct (Muthén and Muthén, 1998–2012; Hoyle, 2012; Brown, 2015; Kline, 2016).

It should be highlighted that, in the reported CFA, item residuals of two different factors were correlated (e.g., item 1 of the exercise factor with item 1 of the diet factor). This practice is commonly discouraged, as it suggests that items of two different factors are not independent (Muthén and Satorra, 1995; Hoyle, 2012; Kline, 2016), and it should be usually considered a “wastebasket” method to improve model fit. However, it is important to highlight that in the case of equally worded items—such as in this case (see Appendix A)—this practice allows these correlations to simply recognize and incorporate the covariance that results from using exactly the same items with different target (Kelloway, 2015). Indeed, when no correlated uniqueness is specified, all of the covariations among item loading on a latent factor are due to that factor—and all measurement errors are random (Brown, 2015). Conversely, in the case of equally worded items, correlated residuals between items of different factors are specified on the basis of the awareness that some of the covariance in the items not explained by the latent factor is attributable to another reason (Brown, 2015), such as the same item wording (Kenny, 1979; Marsh, 1989; Marsh and Bailey, 1991; Kenny and Kashy, 1992; Tomás et al., 2000; Brown, 2015).

However, in order to further test the goodness of the MED-C, statistical analyses were replicated without correlation of the residuals (Supplementary Material S1)—and results confirming the structural validity and reliability of the questionnaire.

Moreover, considering that the MED-C is a new questionnaire, the proposed factorial structure was compared with several possible competing models: a model without correlation of the residuals, a single-factor model, a second-order structure, and a bifactor model. Results of model comparison demonstrated the superiority of the proposed factorial structure.

Item discrimination power was also tested. Results showed that each of the eight items composing the MED-C exercise subscale well discriminated between subjects with low and individuals with high motivation to exercise. Similarly, for the diet subscale, the item discrimination power indicated that each of the eight items composing this factor well discriminated between subjects with low and individuals with high motivation to diet. These results suggest the goodness of the items to discriminate between different types of motivation in the individuals, as well as the ability of each item to represent its latent construct.

Reliability analyses were also performed. Given the originality of the MED-C, and because of possible differences in the magnitude of items’ factor loading (Barbaranelli et al., 2014), MR coefficient was chosen as a measure of internal consistency (Raykov, 2011, 2012; Raykov and Marcoulides, 2011), demonstrating high reliability of both subscales.

Convergent validity analyses were also performed. Small to moderate statistically significant positive correlations were found between both the MED-C subscales (exercise and diet) and all the dimensions of the BFQ-C (0.164–0.400), except for the emotional–instability factor. Notably, the highest correlation (0.400) was observed between the MED-C-exercise and the energy–extraversion subscales. These results indicate a link between personality traits and SDT-internalized degrees of self-regulation, thus supporting conclusions from previous empirical studies that think of causality orientations (autonomy, control, and impersonal) as characteristic adaptations of dispositional traits (Brinkman et al., 2016; Prentice et al., 2019). In other words, the behavior associated with each personality factors would be differentially self-determined. These conclusions highlight the importance for health care professionals to adequately consider the role played by personality factors in motivating children in the initiation and persistence of healthy behaviors (Deci and Ryan, 1990; Ng et al., 2012).

Moreover, the exercise and diet subscales of the MED-C showed positive—albeit weak—statistically significant associations with the presence of symptoms of an eating disorder (KEDS–total score), but no relationship with the occurrence of psychogenic eating disorders (SAFA-P/e–total score)—as expected when recruiting children from the general population. Specifically, convergent relationships were detected between the diet dimension and both the weight dissatisfaction (0.148) and the purging/restricting (0.108) subscales of KEDS, whereas the exercise dimension of the MED-C correlated only with a possible presence of weight dissatisfaction (0.150). These dimensions might therefore represent reasons to exercise and diet in children.

Moreover, when considering the diverse aspects of the eating disorders as means of the SAFA-P/e, small but positive statistically significant correlations were found between perfectionism and the MED-C subscales (exercise: 0.159, diet: 0.130). Instead, trivial to small negative associations were observed between the MED-C dimensions, and both the inadequacy (exercise: −0.095; diet: −0.160) and the bulimic behavior factor (exercise: −0.092; diet: −0.112) of the *SAFA-P/e*. The MED-C-diet subscale also showed low positive statistically significant association with anorexic behavior.

Despite that research suggests SDT to be an interesting theoretical framework to examine motivational processes underlying disordered eating behaviors, previous studies (Pelletier et al., 2004a, b; Pelletier and Dion, 2007) did not include the contribution of basic psychological needs satisfaction in their explanatory model.

The MED-C might overcome this limit, as it represents the first psychometrically sound instrument able to address the lack of relevant data concerning the role of psychological needs satisfaction in the motivational sequence. This measure can therefore be used by clinicians and researchers to promptly assess the problem and to increase autonomous self-regulation according to the specific child’s expressed psychological needs. Needs satisfaction has, in fact, proven association with healthier behaviors (Ryan et al., 2008), whereas unhealthy weight control behaviors (Thogersen-Ntoumani et al., 2010) and symptoms of eating disorders are associated with unmet needs satisfaction (Kopp and Zimmer-Gembeck, 2011; Schüler and Kuster, 2011).

Because central to scale validation is testing the invariance of factorial structures between different population, MI analyses were performed to evaluate at which level (structural vs. loadings vs. thresholds vs. latent means) the MED-C’s item invariance properties lead to similar functioning across gender, age, and perception to have at least one parent with overweight and/or obesity.

In fact, studies have indicated higher parental BMI to be an important predictor for weight gain from childhood to adolescence (13) as a result of complex interaction between genetic and environmental effects (Xi et al., 2009; Bahreynian et al., 2017). Moreover, stable exposure to heavier body weights may alter visual perceptions of what constitutes a “normal” weight in children and shift the visual threshold of what constitutes a normal-weight body in the direction of overweight, thus increasing the likelihood that unhealthy behaviors will be implemented (Robinson, 2017).

Results suggested that the MED-C was invariant across all groups—at least at the thresholds/intercepts level. Considering gender, MI analyses showed that strict invariance was achieved. These results revealed that males and females had the same expected item response at the same absolute level of the trait (strict invariance), but they did not have the same expected latent mean of the traits. Moreover, considering both age (≤10 vs. ≥11 years) and the perception to have at least one parent with overweight and/or obesity (yes vs. no), MI analyses showed that latent means invariance was achieved.

Findings from this study support the reliability of the MED-C questionnaire in measuring motivation to diet/exercise both in children independently from their age, gender, and exposure to heavier or normal body weights.

Therefore, the MED-C questionnaire can be used in research and clinical practice to compare results derived from these groups due to the fact that children interpreted its items in the same way (the factorial structure was equal across groups), with the same strength (items were equally related to the latent construct between the groups), and having the same starting point (item thresholds were equal across groups).

Still, despite that preliminary analyses showed that the MED-C was non-dependent from data clustering and the sample size was considered sufficient to perform selected statistics, the present study did not consider group interactions in the assessment of MI (e.g., ≤9 years male vs. ≥10 years male vs. ≤9 years female vs. ≥10 years female). Future studies should overcome this gap by improving the sample size for each group. Moreover, in this study, a convenience sample of children living in Milan and the surrounding Province was recruited from the general population—and inclusion of research participants from other regions of Italy would extend the ecological validity and generalizability research findings. An independent replication and assessment of the psychometric properties and factorial structure of the MED-C across different populations (clinical vs. non-clinical), countries, and languages are also needed. Further limitations are the exclusive use of self-report measures to test convergent validity. Finally, the data used in this study had a cross-sectional nature, thus not allowing for testing of MED-C changes over time or assessment of its predictive validity (e.g., test–retest reliability and longitudinal MI analysis). Moreover, future studies should consider creating latent psychological profiles in order to identify recurring patterns of motivation to diet and exercise in children—aimed at maintaining healthy behaviors (Hagenaars and McCutcheon, 2006; Huh et al., 2011; Patnode et al., 2011; Mannarini et al., 2013, 2017; Vannucci et al., 2013).

Despite the limitations, this study had the strength of assuming, for the first time, the presence of amotivation, thus testing a more complete motivational continuum.

The parsimony of the assessment would also reduce the time needed to complete the questionnaire without cutting the information obtained, thus minimizing participant burden.

## Conclusion

The findings support the use of the MED-C questionnaire to assess motivational regulation and needs among children in the context of exercise and diet. The MED-C possesses good construct validity and reliability and retains the SDT-based conceptualization of behavioral regulation. Further, research should seek to replicate these findings in clinical or treatment-seeking samples, also employing cross-cultural designs, and examine the MED-C relationship with anthropometrics and metabolic characteristics.

## Data Availability Statement

The raw data supporting the conclusions of this article will be made available by the authors, without undue reservation.

## Ethics Statement

The studies involving human participants were reviewed and approved by IRCCS Istituto Auxologico Italiano Ethics Committee. Written informed consent to participate in this study was provided by the participants’ legal guardian/next of kin.

## Author Contributions

GP, AR, and MB contributed to the conceptualization and methodology. MB collected the data. AR contributed to the data curation and formal analysis. GP and AR wrote the original draft of manuscript. All authors have reviewed and edited the manuscript.

## Conflict of Interest

The authors declare that the research was conducted in the absence of any commercial or financial relationships that could be construed as a potential conflict of interest.

## Appendix

**APPENDIX A:** The Motivation to Exercise and Diet (MED-C) Questionnaire—Children Version.

			Exercise factor	Diet factor
**Motivation**				
1	Intrinsic	IT	Mi piace fare sport	Mi piace mangiare sano
		EN	*I like to do sport*	*I like to eat healthy*
2	Identified	IT	Per me è importante fare sport	Per me è importante mangiare sano
		EN	*It’s important to me to do sport*	*It’s important to me to eat healthy*
3	Introjected	IT	Fare sport mi fa stare bene	Mangiare sano mi fa stare bene
		EN	*Playing sports makes me feel good*	*Eating healthy makes me feel good*
4	External	IT	Gli altri (amici, famigliari) mi dicono che devo fare sport	Gli altri (amici, famigliari) mi dicono che devo mangiare sano
		EN	*The others (friends/family) say I should do sport*	*Other people (friends/family) say I should eat healthy.*
5	Amotivation	IT	Non mi interessa fare sport	Non mi interessa mangiare sano
		EN	*I don’t care about doing sports*	*I don’t care about eating healthy*
**Need**				
6	Autonomy	IT	Posso decidere quale sport fare	Posso decidere cosa mangiare/quali cibi mangiare
		EN	*I can decide which sport to do*	*I can decide what to eat*
7	Competence	IT	Credo di essere bravo/a nello sport	Credo di essere bravo nel seguire una sana alimentazione/dieta sana
		EN	*I think I’m good at sports*	*I think I’m good at following a healthy diet*
8	Relatedness	IT	Fare sport mi fa sentire parte di una squadra	Mangiare insieme agli altri (amici/famigliari) mi fa sentire “in famiglia”
		EN	*Playing sports makes me feel part of a team*	*Eating with others (friends/family) makes me feel “in the family”*

*IT, Italian; EN, English translation.*
