# Supplementary material for: Development and Validation of a Self-Determination Theory-Based Measure of Motivation to Exercise and Diet in Children
Source: Front Psychol. 2020 Jun 30;11:1299. doi: 10.3389/fpsyg.2020.01299 (PMC7340182; doi:10.3389/fpsyg.2020.01299)
Supplement: Supplementary file 1 [file Table_1.DOCX]

**S1. Supplementary material 1:**

**Confirmatory Factor Analysis (CFA) and Measurement Invariance (MI) of the** **MED-C questionnaire - without correlations of the residuals.**

In order to further test the goodness of the MED-C questionnaire statistical analysis (structural validity, model comparisons, and MI, using the same estimator – DWLS) were performed without correlating item residuals (Figure S1-1). Results showed once more the good psychometrical properties of this questionnaire.

***Structural validity***

The MED-C showed a good fit to the data. Despite the Chi-square statistic resulted to be statistically significant [S-Bχ^2^ (103) = 288.593; *p* < 0.001], all the other fit indices revealed a good fit to the data: the CFI = 0.987, the RMSEA = 0.059; 90%CI 0.051–0.067; *p*(RMSEA < 0.05) = 0.033, the WRMR = 1.252. As reported in Table 2, all the items’ loadings were statistically significant and ranged from 0.407 (item#4) to 0.916 (item#3), with a mean equal to 0.702 and a SD equal to 0.172.

Moreover, the two first-order factor solution was compared with different competing models that could also explain the MED-C factorial structure ([Brown, 2015](#_ENREF_1); [Kline, 2016](#_ENREF_2); [Muthén & Muthén, 1998-2012](#_ENREF_3)). As reported in Table S1-1, model comparisons revealed the superiority of the proposed solution: two related first-order factor model – accounting for two different dimensions – without correlation of residuals.

***Measurement Invariance***

*Gender* (male vs. female)

*Configural Invariance*. The configural invariance model showed good model fit indices: S-Bχ^2^ (206) = 397.63, *p* < 0.001; CFI = 0.987; and the RMSEA = 0.060; suggesting that the factor structure was similar between males and females.

*Metric Invariance.* The metric invariance model well fitted the data: S-Bχ^2^ (220) = 405.56, *p* < 0.001; CFI = 0.988; and the RMSEA = 0.057. Non-significant decreases in fit indices were found (DIFTEST (14) = 7.930; *p* = 0.893; |ΔRMSEA| = 0.003; |ΔCFI| = 0.001), indicating that items were equivalently related to the latent factor between males and females.

*Scalar Invariance.* The scalar invariance model showed good model fit indices: S-Bχ^2^ (266) = 451.44, *p* < 0.001; CFI = 0.988; and the RMSEA = 0.052. Non-significant decreases in fit indices were found (DIFTEST (46) = 45.874; *p* = 0.477; |ΔRMSEA| = 0.005; |ΔCFI| = 0.000), suggesting that males and females had the same expected item response at the same absolute level of the trait.

*Latent Means Invariance.* The latent mean invariance model well-fitted the data: S-Bχ^2^ (268) = 537.36, *p* < 0.001; CFI = 0.982; and the RMSEA = 0.062. Non-significant decreases in fit indices were found (DIFTEST (2) = 85.924; *p* < 0.001; |ΔRMSEA| = 0.010; |ΔCFI| = 0.006), suggesting that males and females had the same expected latent mean of the traits.

*Age* (median slit technique: < 10 y.o. *vs.* > 11 y.o.)

*Configural Invariance*. The configural invariance model showed good model fit indices: S-Bχ^2^ (206) = 399.88, *p* < 0.001; CFI = 0.987; and the RMSEA = 0.061; suggesting that the factor structure was similar between different age-related groups.

*Metric Invariance.* The metric invariance model well-fitted the data: S-Bχ^2^ (220) = 435.96, *p* < 0.001; CFI = 0.986; and the RMSEA = 0.062. Non-significant decreases in fit indices were found (DIFTEST (14) = 36.083; *p* = 0.001; |ΔRMSEA| = 0.001; |ΔCFI| = 0.001), indicating that items were equivalently related to the latent factor between groups.

*Scalar Invariance.* The scalar invariance model showed good model fit indices: S-Bχ^2^ (266) = 517.44, *p* < 0.001; CFI = 0.984; and the RMSEA = 0.060. Non-significant decreases in fit indices were found (DIFTEST (46) = 81.477; *p* < 0.001; |ΔRMSEA| = 0.001; |ΔCFI| = 0.002), suggesting that the two groups had the same expected item response at the same absolute level of the trait.

*Latent Means Invariance.* The latent mean invariance model showed good model fit indices: S-Bχ^2^ (268) = 531.34, *p* < 0.001; CFI = 0.983; and the RMSEA = 0.062. Non-significant decreases in fit indices were found (DIFTEST (2) = 13.895; *p* < 0.001; |ΔRMSEA| = 0.001; |ΔCFI| = 0.001), suggesting that the two had the same expected latent mean of the traits.

*Perception to have at least one parent with overweight or obesity* (yes *vs.* no)

*Configural Invariance*. The configural invariance model showed good model fit indices: S-Bχ^2^ (206) = 399.36, *p* < 0.001; CFI = 0.987; and the RMSEA = 0.060; suggesting that the factor structure was similar between children who perceived a parent with overweight/obesity and children who did not.

*Metric Invariance.* The metric invariance model still fitted data well: S-Bχ^2^ (220) = 408.22, *p* < 0.001; CFI = 0.987; and the RMSEA = 0.057. Non-significant decreases in fit indices were found (DIFTEST (14) = 8.869; *p* = 0.840; |ΔRMSEA| = 0.003; |ΔCFI| = 0.000), indicating that items were equivalently related to the latent factor between groups.

*Scalar Invariance.* The scalar invariance showed good model fit indices: S-Bχ^2^ (266) = 458.03, *p* < 0.001; CFI = 0.987; and the RMSEA = 0.053. Non-significant decreases in fit indices were found (DIFTEST (46) = 49.805; *p* = 0.324; |ΔRMSEA| = 0.004; |ΔCFI| = 0.000), indicating that items were equivalently related to the latent factor irrespectively of children’s perception to have at least one parent with overweight/obesity and their counterpart.

*Latent Means Invariance.* The latent mean invariance model still fitted data well: S-Bχ^2^ (268) = 458.64, *p* < 0.001; CFI = 0.987; and the RMSEA = 0.052. Non-significant decreases in fit indices were found (DIFTEST (2) = 0.614; *p* = 0.736; |ΔRMSEA| = 0.001; |ΔCFI| = 0.000), suggesting that the two groups had the same expected latent mean of the traits.


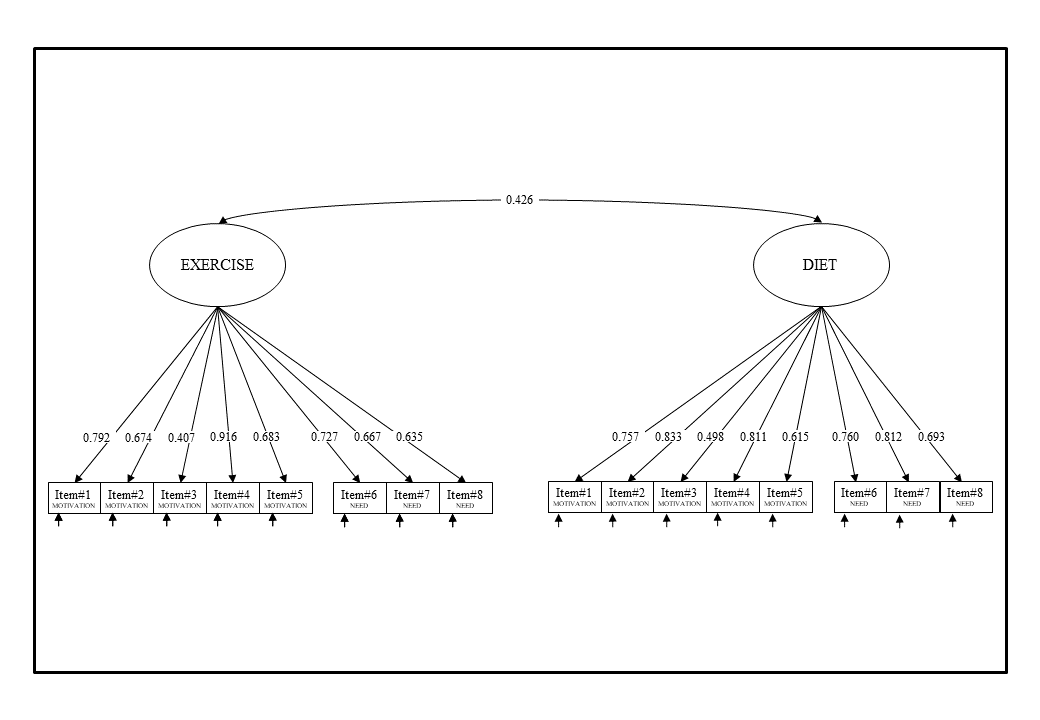
*Figure S1-1*. Graphical representation of the CFA.

*Table S1-1.* Model Comparison.

|  | S-Bχ2 (*df*) | RMSEA | CFI | Comparison | DIFF-TEST | \|ΔRMSEA\| | \|ΔCFI\| |
| --- | --- | --- | --- | --- | --- | --- | --- |
| *Model 1*: two related factors – no correlation of residuals | 288.593*** (103) | 0.059 | 0.987 |  |  |  |  |
| *Model 2*: single factor model | 1683.750*** (104) | 0.171 | 0.889 | 2 vs 1 | 1395.2*** | 0.112 | 0.098 |
| *Model 3*: two independent factor model | 1534.712*** (104) | 0.163 | 0.899 | 3 vs 1 | 1246.10*** | 0.104 | 0.088 |
| *Model 4*: second order model | Not identified | - | - | 4 vs 1 | - | - | - |
| *Model 5*: bi-factor model | No convergence | - | - | 5 vs 1 | - | - | - |

§*p* > 0.050 *ns*; **p* < 0.050; ***p* < 0.010; ****p* < 0.001. S-Bχ2 = Satorra-Bentler scaled chi-square test; *df* = degrees of freedoms; |Δ(…)| = absolute value of the differences between indices; RMSEA = root mean square error of approximation; CFI = comparative fit index.

*Table S1-2*. Measurement Invariance of the MED-C – without correlations of the residuals.

|  | S-Bχ2 (*df*) | RMSEA | CFI |  | DIFF-TEST | *p*(DIFFTEST) | \|ΔRMSEA\| | \|ΔCFI\| |
| --- | --- | --- | --- | --- | --- | --- | --- | --- |
| GENDER |  |  |  |  |  |  |  |  |
| Model ‘Male’ (*n* = 285) | 150.77* (103) | 0.043 | 0.995 |  |  |  |  |  |
| Model ‘Female’ (*n* = 305) | 246.89*** (103) | 0.072 | 0.976 |  |  |  |  |  |
| Configural Inv. | 397.63*** (206) | 0.060 | 0.987 |  |  |  |  |  |
| Metric Inv. | 405.56*** (220) | 0.057 | 0.988 |  | 7.930 (14) | *p* = 0.893 | 0.003 | 0.001 |
| Strict Inv. | 451.44*** (266) | 0.052 | 0.988 |  | 45.874 (46) | *p* = 0.477 | 0.005 | 0.000 |
| Mean Inv. | 537.36*** (268) | 0.062 | 0.982 |  | 85.924 (2) | *p* < 0.001 | 0.010 | 0.006 |
|  |  |  |  |  |  |  |  |  |
| AGE |  |  |  |  |  |  |  |  |
| Model ‘< 10 y.o.’ (*n* = 269) | 209.41*** (103) | 0.066 | 0.983 |  |  |  |  |  |
| Model ‘> 11 y.o.’ (*n* = 321) | 109.47*** (103) | 0.055 | 0.991 |  |  |  |  |  |
| Configural Inv. | 399.88*** (206) | 0.061 | 0.987 |  |  |  |  |  |
| Metric Inv. | 435.96*** (220) | 0.062 | 0.986 |  | 36.083 (14) | *p* = 0.001 | 0.001 | 0.001 |
| Strict Inv. | 517.44*** (266) | 0.060 | 0.984 |  | 81.477 (46) | *p* < 0.001 | 0.001 | 0.002 |
| Mean Inv. | 531.34*** (268) | 0.062 | 0.983 |  | 13.895 (2) | *p* < 0.001 | 0.001 | 0.001 |
|  |  |  |  |  |  |  |  |  |
| PARENT(s) WITH OBESITY |  |  |  |  |  |  |  |  |
| Model ‘YES’ (*n* = 225) | 168.24*** (103) | 0.056 | 0.988 |  |  |  |  |  |
| Model ‘NO’ (*n* = 365) | 231.12*** (103) | 0.062 | 0.986 |  |  |  |  |  |
| Configural Inv. | 399.36*** (206) | 0.060 | 0.987 |  |  |  |  |  |
| Metric Inv. | 408.22*** (220) | 0.057 | 0.987 |  | 8.869 (14) | *p* = 0.840 | 0.003 | 0.000 |
| Strict Inv. | 458.03*** (266) | 0.053 | 0.987 |  | 49.805 (46) | *p* = 0.324 | 0.004 | 0.000 |
| Mean Inv. | 458.64*** (268) | 0.052 | 0.987 |  | 0.614 (2) | *p* = 0.736 | 0.001 | 0.000 |

§*p* > 0.050 *ns*; **p* < 0.050; ***p* < 0.010; ****p* < 0.001. S-Bχ2 = Satorra-Bentler scaled chi-square test; *df* = degrees of freedoms; |Δ(…)| = absolute value of the differences between indices; RMSEA = root mean square error of approximation; CFI = comparative fit index.

**References**

Brown, T. A. (2015). *Confirmatory Factor Analysis for Applied Research* (Second ed.). New York: The Guilford Press.

Kline, R. B. (2016). *Principles and practice of structural equation modeling*. New York: The Guilford Press.

Muthén, L. K., & Muthén, B. O. (1998-2012). *Mplus User’s Guide* (Seventh Edition ed.). Los Angeles, CA.
