# Supplementary material for: Development and Validation of a Self-Determination Theory-Based Measure of Motivation to Exercise and Diet in Children
Source: Front Psychol. 2020 Jun 30;11:1299. doi: 10.3389/fpsyg.2020.01299 (PMC7340182; doi:10.3389/fpsyg.2020.01299)
Supplement: Supplementary file 2 [file Table_2.DOCX]

**S2. Supplementary material 2:**

**Confirmatory Factor Analysis (CFA) and Measurement Invariance (MI) of the MED-C questionnaire - without correlations of the residuals.**

In order to further explore the structural validity of the MED-C, a CFA was run with a second alternative model of the instrument intended to address the systematic variance due to wording valence (motivation and need) while maintaining the distinction between *exercise* and *diet* ([Thompson et al., 2005](#_ENREF_1)). Two latent factors for ‘motivation’ and ‘need’ valence were indicated by the motivation (from item#1 to item item#5; of each dimension – *exercise* and *diet*) and need (from item#6 to item item#8; of each dimension – *exercise* and *diet*) worded item, respectively. These valence factors were correlated following the self-determination theory (SDT) conceptualization.

Even in this case, results confirmed the good psychometrical properties of the questionnaire.

No further analysis was run on this model. In fact, SDT recognizes the role of psychological need satisfaction in underpinning different types of motivation. These dimensions are therefore statistically related yet theoretically distinct components of behavioural regulation. Moreover, clinicians and researchers are interested in the quality, in addition to the quantity, of motivation for a target behavior.

The alternative model of the MED-C does not allow the simultaneous recognition of the different motivational regulation and needs of the individual for two dimensions (exercise and diet) separately, while the original factor structure of the questionnaire does.

***Structural validity***

The MED-C showed a good fit to the data. Despite the Chi-square statistic resulted to be statistically significant [χ^2^ (102) = 360.684 *p* < 0.001], all the other fit indices revealed a good fit to the data: the CFI = 0.960, the RMSEA = 0.066; 90%CI 0.058–0.073; *p*(RMSEA < 0.05) < 0.001, the WRMR = 1.242.

*Figure S2-1*. Graphical representation of the CFA


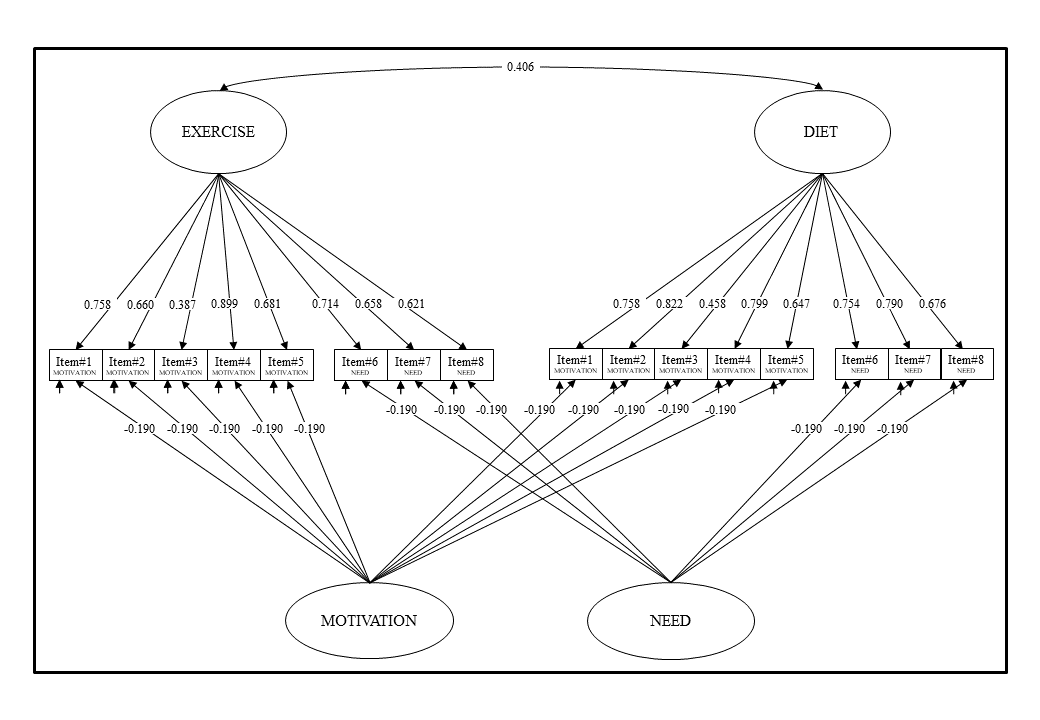


**References**

Thompson, L. Y., Snyder, C. R., Hoffman, L., Michael, S. T., Rasmussen, H. N., Billings, L. S., . . . Roberts, D. E. (2005). Dispositional forgiveness of self, others, and situations. [Research Support, Non-U.S. Gov't]. *J Pers, 73*(2), 313-359. doi: 10.1111/j.1467-6494.2005.00311.x
